# Inter- and intraobserver variability in motor mapping of the hotspot for the abductor policis brevis muscle

**DOI:** 10.1186/1471-2202-14-94

**Published:** 2013-09-05

**Authors:** Nico Sollmann, Theresa Hauck, Thomas Obermüller, Alexander Hapfelmeier, Bernhard Meyer, Florian Ringel, Sandro M Krieg

**Affiliations:** 1 Department of Neurosurgery, Klinikum rechts der Isar, München, Germany; 2 Institute of Medical Statistics and Epidemiology Technische Universität München, Ismaninger Str. 22, 81675, Munich, Germany

**Keywords:** Cortical mapping, Transcranial magnetic stimulation, Motor mapping, Navigated brain stimulation, Motor threshold, Hand knob

## Abstract

**Background:**

For accuracy in navigated transcranial magnetic stimulation (nTMS), determination of the hotspot location of small hand muscles is crucial because it is the basis for the resting motor threshold (RMT) and, therefore, its spatial resolution. We investigated intra- and interobserver differences of hotspot mapping to provide evidence for the reproducibility of this method.

Ten subjects underwent nTMS motor mapping of the hotspot for the abductor pollicis brevis muscle (APB) three times. The first two sessions were performed by the same examiner; the third mapping was performed by a different examiner. Distances between the first and second mappings (intraobserver variability) and between the second and third mappings (interobserver variability) were measured.

**Results:**

Intraobserver variability had a mean of 8.1 ± 3.3 mm (limits of agreement (LOA) 1.7 to 14.6 mm), whereas mean interobserver variability was 10.3 ± 3.3 mm (LOA 3.8 to 16.7 mm). Concerning RMT, CCC was 0.725 (95% CI: 0.276; 0.914). The mean variability in the same cortical depth was measured as 5.7 ± 3.3 mm (LOA −0.7 to 12.2 mm) for intraobserver and 9.2 ± 3.3 mm (LOA 2.7 to 15.8 mm) for interobserver examinations. When evaluating the RMT, CCC was 0.709 (95% CI: 0.244; 0.909).

**Conclusions:**

Overall, intraobserver variability showed higher reliability than interobserver variability. Our findings show that we can achieve good reliability in hotspot determination, ranging within the calculated precision of the system.

## Background

In recent years, navigated transcranial magnetic stimulation (nTMS) has been increasingly competing with other diagnostic modalities in visualizing functional brain areas [1]. Having started as an approach for determining cortical motor areas, this promising technique has developed into a multifunctional tool for various diagnostic and therapeutic issues. Although intraoperative direct cortical stimulation (DCS) is the gold standard for the mapping of brain functions, it is not able to provide exact information about the healthy human brain, mainly because of its invasiveness. One main benefit of nTMS is its non-invasive nature, which allows the implementation of this method in the preoperative setup [2]. Additionally, a correlation of nTMS and DCS in motor mapping has already been reported [1,3-5]. However, until now there have only been a few studies on the reproducibility of nTMS motor mapping and individual resting motor threshold (RMT), especially concerning intra- and interobserver investigations. Former studies showed comparable results in repeated mappings. However, these data is based on non-navigated TMS [6].

Finding the same spot for determination of the RMT seems crucial because the RMT determines spatial resolution of nTMS motor mapping. And despite various influencing factors which might impair the measurement, RMT should be determined at the same cortical spot. Even when this claim does not mirror reality. Knowledge about hotspot location stability in repeated nTMS investigations is essential in detecting the correct RMT. Inaccuracy in this step at the beginning of the mapping session causes imprecision in the whole procedure.

Moreover, besides preoperative nTMS motor mapping, recent data show that TMS also serves as a neurophysiological assessment of the functional status of the patient’s motor system especially for neurorehabilitation issues [7].

As the reliability of the hotspot location is crucial for the accuracy of nTMS examinations, this study provides data about intra- and interobserver variability in motor mapping of the hotspot for the abductor pollicis brevis muscle (APB).

## Methods

### Study design

Ten healthy subjects underwent nTMS motor mapping of the hotspot for the right APB on the left hemisphere. This examination was performed three times at three different days. The first and second mappings were conducted by the same investigator in order to evaluate intraobserver variability. Additionally, interobserver variability was determined by comparing the second mapping and a third mapping performed by a different examiner. Both investigators were blinded to previous results and their experience in nTMS was comparable. Moreover, both investigators had considerable experience in nTMS mapping prior to this study.

### Subjects

The volunteers suffered from no cerebral pathology and were all right-handed. Five subjects were male, five subjects were female. The median age was 24.2 years (range 22.7 to 30.3 years). No one was under any kind of medication.

### Ethical standard

The study was conducted with the consent of the local ethics committee of the Technical University of Munich (registration number: 2793/10) and in accordance with the Declaration of Helsinki. Written informed consent was obtained from all volunteers prior to navigational MRI.

### MRI acquisition

All subjects underwent MR imaging prior to the first mapping. MRI was performed on a 3 Tesla MR scanner combined with an 8-channel phased array head coil (Achieva 3 T, Philips Medical Systems, The Netherlands B.V.). The scanning protocol consisted of a 3D gradient echo sequence (TR/TE 9/4 ms, 1 mm^2^ isovoxel covering the whole head, 6 min 58 s acquisition time) without intravenous contrast administration for anatomical co-registration. Using DICOM standards, the 3D dataset was then transferred to the nTMS system.

### Navigated transcranial magnetic stimulation

At each mapping session, the subjects underwent the same procedure for mapping of the hotspot as described in earlier reports [3,5]. In short, the used nTMS system (eXimia 4.3, Nexstim, Helsinki, Finland) includes a magnetic stimulator with a biphasic figure-of-eight TMS coil with a radius of 50 mm. The navigation device (Polaris Spectra, Waterloo, Ontario, Canada) orients individual 3D MR images to the patient’s head by infrared tracking using spheres coated with retro-reflective surface. The used nTMS system estimates the induced electric field strength with regard to the patient’s head shape and then visualizes this electric field strength on the cortical surface, which is reconstructed by the MRI dataset [8,9]. While stimulating with nTMS, electromyography (EMG) (eXimia 4.3, Nexstim, Helsinki, Finland) is triggered by nTMS stimuli and monitored continuously. EMG was recorded over the skin of the APB by pregelled disposable Ag/AgCl electrodes (Neuroline 720, Ambu, Bad Nauheim, Germany) and the reference electrode was placed at the ipsilateral elbow above the tendon of the biceps muscle. The sampling rate was 3 kHz; resolution was 0.3 μV. Noise of the device was lower than 5 μV for peak-to-peak measurements. While applying stimulation over the left-hemispheric motor cortex, motor evoked potentials (MEP) of the right APB were measured by EMG. The subjects were told to focus on the EMG to relax their muscles. Thus, we achieved a highly comparable alertnes.

The stimulation intensity was adjusted that the EMG response was between 100 and 600 μV. We detected the most excitable site in the anatomically defined hand knob [10] that elicited the maximum MEP response, commonly defined as the “hotspot” which means that this is the point with the highest MEP amplitude for the right APB after single pulse nTMS. The coil was positioned perpendicular to the course of the central sulcus. For the hotspot determination between 15 and 57 stimuli were administered (Table 1). Afterwards, we performed the individual RMT determination, which was defined as the lowest stimulation intensity that evokes a response in the relaxed APB muscle of more than 50 μV of amplitude in 5 out of 10 stimulations. This protocol was used in all subjects.

**Table 1:** Mapping characteristics

	**1st mapping**	**2nd mapping**	**3rd mapping**	**p**
Median number of stimuli (range)	33 (15 to 57)	35 (17 to 56)	36.5 (19 to 45)	0.9919
Median RMT (% Output) (range)	38.5% (26% to 47%)	38.0% (26% to 44%)	38.0% (24% to 44%)	0.781

This table shows the mapping characteristics of all subjects and stimulation parameters used in the study. RMT = resting motor threshold (stimulator output).

### Data export and measurement

**Figure 1:**
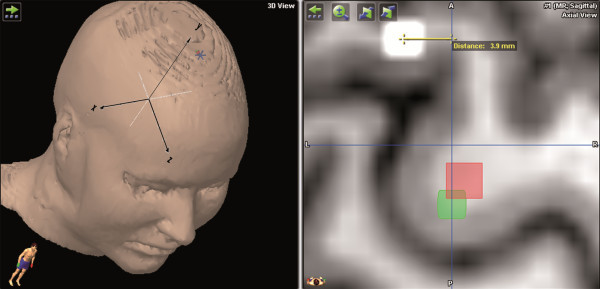
**This figure shows an example of the measurement for hotspot distances.** The yellow line indicates the distance between the white and red spots and, therefore, shows the interobserver hotspot distance in mm (green spot = hotspot first mapping; white spot = hotspot second mapping/first remapping; red spot = hotspot third mapping/second remapping). The left upper quadrant shows the orientation of the used coordinate system.

The detected hotspots were then exported via DICOM standards and imported to the iPlan Net (BrainLAB AG, Feldkirchen, Germany). Image fusion of the three corresponding maps was performed with each subject’s MRI data. We then determined the distances between the hotspots of each mapping in all 3 axes (i.e., x-, y-, and z-axes) (Figure 1).

### Statistical analysis

The distribution of continuous data is summarized by mean ± standard deviation (SD) and presented by boxplots. Comparisons of intra- and interobserver distances are performed by paired-samples t-tests. The variability of the different mappings was evaluated and visualized by a Bland-Altman plot [1]. When regarding the hotspot distances between the different measurements, not only mean ± SD but also limits of agreement (LOA) (= mean ± 1.96 × SD) were calculated which represent the 95% likely range for the difference between two measurements. Moreover, to calculate concordance of the RMT, Lin’s concordance correlation coefficient (CCC) as well as its 95% confidence interval (95% CI) was calculated. Concerning RMT, differences between groups were tested by the Kruskall-Wallis test for nonparametric one-way analysis of variance (ANOVA) followed by Dunn’s test as the post hoc test. All statistical tests were conducted in an explorative manner on a two-sided 5% significance level.

## Results

### nTMS parameters

During the initial mapping, the median RMT for the left hemisphere was defined as 38.5% (range 26.0% to 47.0%) of the maximal output. The second mapping (i.e., first remapping) was conducted with a left-hemispheric RMT of 38.0% (range 26.0% to 44.0%) of the system’s output. During the third mapping (i.e., second remapping), the RMT for the left hemisphere was determined as 38.0% (range 24.0% to 44.0%) of the maximal output (Table 1).

### Intraobserver variability

**Figure 2:**
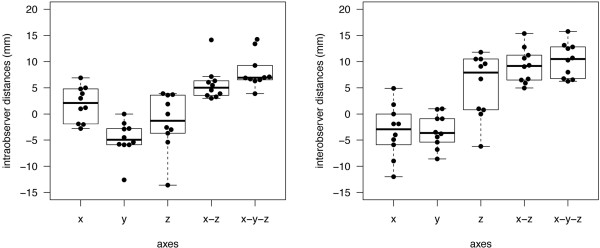
The boxplot shows intraobserver (left side) and interobserver (right side) distances for x-, y-, z-, as well as for x-z- and x-y-z-coordinates.

**Figure 3:**
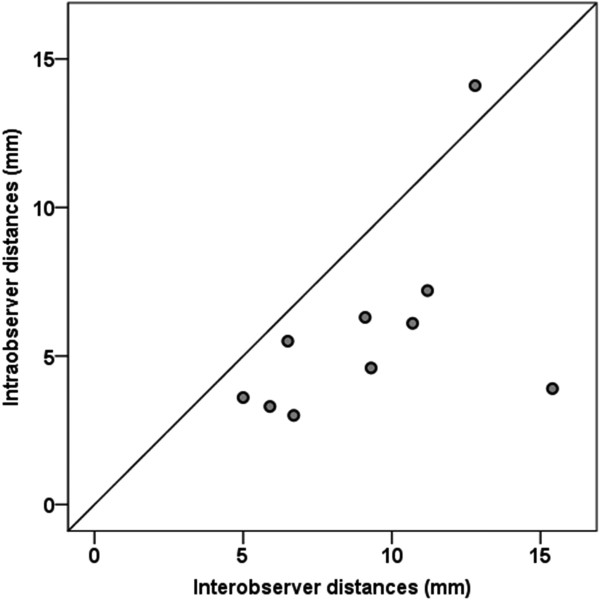
**This figure illustrates the spread of intraobserver (y-axis) and interobserver (x-axis) distances of the hotspots in one peeling depth (x-z-coordinates; *p*** **= 0.010).**

**Figure 4:**
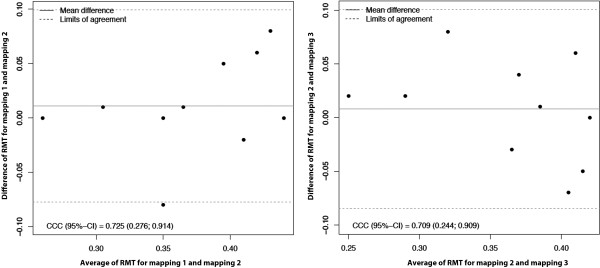
Variability of the RMT of the different mappings evaluated and visualized by a Bland-Altman plot for intraobserver (left side) and interobserver (right side) measurements.

Intraobserver hotspot distances were defined as the difference between the initial and the second mapping (i.e., first remapping). The mean difference of x-coordinates (medio-lateral direction) was measured as 1.9 ± 3.4 mm (LOA −4.7 to 8.5), whereas the mean difference between y-coordinates (cranio-caudal direction) was −4.8 ± 3.4 mm (LOA −11.4 to 1.9; Figure 2). Additionally, the resulting distance between both hotspots was measured as −1.5 ± 5.4 mm (LOA −12.2 to 9.1) for the z-coordinate (anterior-posterior direction). Taking into account not only one direction, the mean distance between x- and z-coordinates—that is, the distance in the same cortical depth (combined x- and z-axis)—was 5.7 ± 3.3 mm (LOA −0.7 to 12.2; Figures 2 and 3). The mean three-dimensional distance (all axes) between hotspots was 8.1 ± 3.3 mm (LOA 1.7 to 14.6; Figure 2). Concerning RMT, CCC was 0.725 (95% CI: 0.276; 0.914) (Figure 4).

### Interobserver variability

Interobserver hotspot distances were defined as the distance between the second mapping (i.e., first remapping) and the third mapping (i.e., second remapping), which was performed by a different examiner, as mentioned above. Focusing on x-coordinates (medio-lateral direction), the hotspot distances were measured to be −3.3 ± 5.0 mm (LOA −13.1 to 6.5), whereas the distance between y-coordinates (cranio-caudal direction) was −3.2 ± 3.2 mm (LOA −9.6 to 3.1). Additionally, the distance between hotspots was measured to be 5.4 ± 6.1 mm (LOA −6.5 to 17.3) for the z-coordinate (anterior-posterior direction). For the interobserver comparison, the mean hotspot distance in the same peeling depth (combined x- and z-axis) was 9.2 ± 3.3 mm (LOA 2.7 to 15.8; Figures 2 and 3). The interobserver distance of all combined axes was 10.3 ± 3.3 mm (LOA 3.8 to 16.7; Figure 2). When evaluating the RMT, CCC was 0.709 (95% CI: 0.244; 0.909) (Figure 4).

### Comparison of the measured intra- and interobserver distances

Table 1 shows the properties of the three mappings. In all categories, differences were observed to be not significant. Considering the intra- and interobserver distances between y-coordinates and the three dimensional distances (all axes), we were not able to show any significant differences (y-coordinates: *p* = 0.294, all axes: *p* = 0.156; Figure 2). In contrast, comparing the intra- and interobserver distances with regard to x- and z-coordinates and to the hotspot distances in the same peeling depth (combined x- and z-axes), we observed statistically significant large interobserver distances (x-coordinates: *p* = 0.015; z-coordinates: *p* = 0.023, combined x- and z-axes: *p* = 0.010; Figures 2 and 3).

## Discussion

RMT determination at the hotspot of small hand muscles is considered a measure for motor cortex excitability. Thus, variability of the hotspot plays an important role for nTMS, both in motor and in language mapping, because it determines the accuracy of nTMS investigations.

In this study, still considering the low sample size, intraobserver comparison showed a higher precision than interobserver investigations in motor mapping of the hotspot for the APB (Figures 2 and 3). This difference is statistically significant when comparing the measured distances of both samples with regard to x- and z-coordinates and to the hotspot distances in the same peeling depth. In contrast, considering variations in distances between y-coordinates and between all axes, we were not able to show statistically significant differences (Figure 2). This correlates well with the fact that the peeling depth is adjusted by varying the y-coordinates. It can be modified several millimeters without having a crucial effect on the mapping procedure; thus, its setting can vary within a small range (Figure 2). Yet, we have to keep in mind that the small sample size still minors the power of statistical tests and therefore judgment of our findings.

However, we have to question why interobserver variability is larger than intraobserver variability. Previous studies have shown that there are individual differences among humans concerning not only hotspot localization but also hotspot shapes [11]. Unfortunately, we still do not know much about interindividual variations in measurement of hotspot size.

However, Pascual-Leone, Wassermann et al. (1995) found that the size of the motor areas in the brain for the hand muscles are able to enlarge significantly after only hours of intensive use. Thus, the timing can play an important role in plasticity investigations, especially when the enrolled subjects of our study are university students who have greater than average use of the right hand.

We also compared the RMTs of all three sessions. Previous studies have shown that the value of the individual motor threshold varies [12]. In our findings, the maximum individual fluctuation was 9% of the total output. Nevertheless, on average, the determined RMTs were almost stable and there was only minimal variation from mapping to mapping (Table 1, Figure 4). As mentioned above, no subject was under any kind of medication. Moreover, special attention was paid on comparable alertness during all mappings.

There already exist several studies on mainly non-navigated TMS that deal with reliability in hotspot determination [6,13-18]. Wolf et al. [19], for example, achieved a distance of 8.9 mm between two sessions with a range from 0 to 22.4 mm. They used the hotspot of the extensor digitorum communis muscle instead of the APB. Nevertheless, comparison among these studies is only possible to a certain extent because previous investigations were conducted using a different muscle, a different navigation system, or non-navigated TMS.

The most important error source in non-navigated TMS is inexact coil positioning because minor movements of the coil or modifications of the angle can cause significant deviations in field strength and stimulus location [20]. With the development of nTMS, we are able to reduce this major error source, and respectable accuracy can be attained [8,21]. Yet, the nTMS system is composed of several specific units, such as MRI-registration, infrared camera system, stimulation coil, induced E-field calculation, head shape, head tracker, etc., which all add minor inaccuracies to the calculated overall error of the used system. Thus, the accuracy of the system depends on the accuracy and the interaction of such principal factors. As Ruohonen and Karhu (2010) described in detail, the E-field computation model causes the highest inaccuracy (3.8 mm) in the system, followed by shifting of the head tracker (3.1 mm), and imperfect alignment between anatomical MR images and the individual’s head (2.5 mm) [21]. The localization of the coil constitutes only 1.6 mm deviation.

In the end, the median overall error of the whole system was calculated to be 5.73 mm [21]. The mean distance of all combined axes was 8.1 mm in our intraobserver investigations and 10.3 mm in our interobserver comparisons. Taking into account that the calculated system error impairs both measurements, the inaccuracy of the whole system can increase up to 11.46 mm (2 × 5.73 mm) in the three-dimensional space. Both the mean intra- and the interobserver distances were within this 11.46 mm value, which means, on the one hand, that the two compared hotspots might actually be located at exactly the same spot, although we measured a distance of several millimeters. On the other hand, the smallest determined distance of 3.0 mm could be much larger in reality.

## Conclusions

In other words, our results are within the calculated error of the whole system, and reproducibility seems to be independent of the examiner, at least concerning the nTMS system used in this study. With regard to our findings, we can therefore state that the intraobserver and the interobserver hotspot distances show sufficient reliability within the system’s accuracy for neurosurgical applications.

## Abbreviations

APB: Abductor pollicis brevis muscle; CCC: Concordance correlation coefficient; CI: Confidence interval; DCS: Direct cortical stimulation; EMG: Electromyography; LOA: Limits of agreement; MEP: Motor evoked potentials; MRI: Magnetic resonance imaging; nTMS: Navigated transcranial magnetic stimulation; RMT: Resting motor threshold; SD: Standard deviation.

## Competing interests

The authors declare that they have no competing interests. The study was completely financed by institutional grants from the Department of Neurosurgery and the authors declare that they have no conflict of interest affecting this study. The authors report no conflict of interest concerning the materials or methods used in this study or the findings specified in this paper.

## Authors’ contributions

NS and TH were responsible for data acquisition and drafted the manuscript. TO was responsible for data acquisition and approved and corrected the final version of the manuscript. AH was responsible for statistical analyses, read and approved the final manuscript. BM approved and corrected the final version of the manuscript. FR is responsible for the original idea, the concept, design, and statistical analyses. FR revised the manuscript, approved and corrected the final version. SK handled the acquired data and performed literature research as well as statistical analyses. SK drafted the manuscript and its final revision. SK is also responsible for concept and design. All authors read and approved the final manuscript.

## Authors’ information

NS and TH are medical student who are performing a considerable number of TMS studies. All other authors are strongly involved in the treatment of brain tumors including awake surgery, preoperative mapping, and intraoperative neuromonitoring in a specialized neurooncological center. BM is chairman and FR is vice chairman of the department.
